# Novel insights in dimethyl carbonate-based extraction of polyhydroxybutyrate (PHB)

**DOI:** 10.1186/s13068-020-01849-y

**Published:** 2021-01-07

**Authors:** Beatrice Mongili, Annalisa Abdel Azim, Silvia Fraterrigo Garofalo, Esperanza Batuecas, Angela Re, Sergio Bocchini, Debora Fino

**Affiliations:** 1 Department of Science and Applied Technology (DISAT), Polytechnic University of Turin, Corso Duca degli Abruzzi 24, 10129 Turin, Italy; 2 Centre for Sustainable Future Technology (CSFT), Italian Institute of Technology, Via Livorno 60, 10144 Turin, Italy; 3 Thermal and Fluid Engineering Department, Carlos III University of Madrid, Avenida de la Universidad 30, 28911 Leganés, Madrid Spain

**Keywords:** Polyhydroxybutyrate, Extraction yield, Solvent-based extraction, Dimethyl carbonate, Multifactorial experiments, LCA analysis

## Abstract

**Background:**

Plastic plays a crucial role in everyday life of human living, nevertheless it represents an undeniable source of land and water pollution. Polyhydroxybutyrate (PHB) is a bio-based and biodegradable polyester, which can be naturally produced by microorganisms capable of converting and accumulating carbon as intracellular granules. Hence, PHB-producing strains stand out as an alternative source to fossil-derived counterparts. However, the extraction strategy affects the recovery efficiency and the quality of PHB. In this study, PHB was produced by a genetically modified *Escherichia coli* strain and successively extracted using dimethyl carbonate (DMC) and ethanol as alternative solvent and polishing agent to chloroform and hexane. Eventually, a Life Cycle Assessment (LCA) study was performed for evaluating the environmental and health impact of using DMC.

**Results:**

Extraction yield and purity of PHB obtained via DMC, were quantified, and compared with those obtained via chloroform-based extraction. PHB yield values from DMC-based extraction were similar to or higher than those achieved by using chloroform (≥ 67%). To optimize the performance of extraction via DMC, different experimental conditions were tested, varying the biomass state (dry or wet) and the mixing time, in presence or in absence of a paper filter. Among 60, 90, 120 min, the mid-value allowed to achieve high extraction yield, both for dry and wet biomass. Physical and molecular dependence on the biomass state and solvent/antisolvent choice was established. The comparative LCA analysis promoted the application of DMC/ethanol rather than chloroform/hexane, as the best choice in terms of health prevention. However, an elevated impact score was achieved by DMC in the environmental-like categories in contrast with a minor contribution by its counterpart.

**Conclusion:**

The multifaceted exploration of DMC-based PHB extraction herein reported extends the knowledge of the variables affecting PHB purification process. This work offers novel and valuable insights into PHB extraction process, including environmental aspects not discussed so far. The findings of our research question the DMC as a *green solvent*, though also the choice of the antisolvent can influence the impact on the examined categories.

## Background

Plastics meet countless needs in our daily life, with their applications in packaging, building and constructions, automotive, household, labware, hospital equipment and electronic to cite only some examples. Current plastic production is about 348 million tonnes to answer the market demand, and this number will double in the next decade on the base of market projection [1]. Plastics are petroleum-derived materials and represent a vast source of greenhouse gasses (GHG) emissions during all their life cycles. The rising demand for plastic materials will increment the global emission release up to 6.850 Gt of CO_2_-equivalent by 2050 [2]. Hence the production and consumption of fossil-based plastics must be reduced to leave the place to more sustainable materials. Beyond recycling plastics and using renewable energy for plastics generation, bio-based polymers embody an alternative to conventional plastics. Indeed, if about the 70% of plastic demand would be fulfilled with bio-based plastic, 241–316 Mt of CO_2_-equivalent would be avoided [3]. Polyhydroxyalkanoates (PHAs) are biologically produced plastic-like material acting as a sink of carbon and reducing equivalent for some microbial species [4, 5]. Currently, more than 90 genera of bacteria and some haloarchaea species have been identified as PHAs producers [6]. PHA general structure consists of a monomer of 3-hydroxy fatty acids, where the residual group length can vary between C_1_ to C_14_ [7]. PHAs are completely compostable and biocompatible, resistant to hydrolysis, UV irradiation, insoluble in water and controllable in their thermal and mechanical behaviour and prone to versatile functionalization [8–10]. These properties make PHAs central precursors supportive for the creation of tailor-made products in manifold applications [7]. Among the different types of PHAs, poly-3-hydroxybutyrate (PHB) is a short chain polymer hosting a methyl group at C3 position, which confers to the polymer high crystallinity and rigidity. As PHB is stored within the microbial cytoplasm as granules (0.12 to 0.5 μm), the polymer recovery represents a technological barrier to its application and a high-cost procedure, which accounts for 50% of the final polymer price. Therefore, many efforts have been employed in the extraction and purification processes, among which solvent-based extraction is the most used recovery strategy. Chloroform is the most applied solvent because of its high PHB extraction power. Several studies report values of recovery up to 95% [11–14]. The addition of hexane as anti-solvent improved PHB purity and recovery as reported by Fei et al. [15]. However, the use of those two toxic chemicals negatively impacts on human and environmental protection. Therefore, it is worth seeking alternative chemicals and extraction strategies, which could achieve satisfactory balance in terms of safety and efficiency. Among those, dimethyl carbonate (DMC) has become increasingly important in the chemical industry, mainly because of its versatility as reagent and solvent, and its low toxicity for human health [16, 17]. DMC is an acyclic alkyl carbonate industrially produced by catalytic oxidative carbonylation of methanol through a green process developed by Enichemand UBE Industries (JP) [18]. This solvent is fully biodegradable, non-irritating and non-mutagenic either by contact or by inhalation. DMC owns negligible reactivity in the formation of photochemical smog. Hence it has been excluded from the list of volatile organic compounds [19, 20]. For all the above-mentioned reasons, DMC has been selected as an alternative solvent to chloroform commonly applied for PHB extraction.

**Fig. 1:**
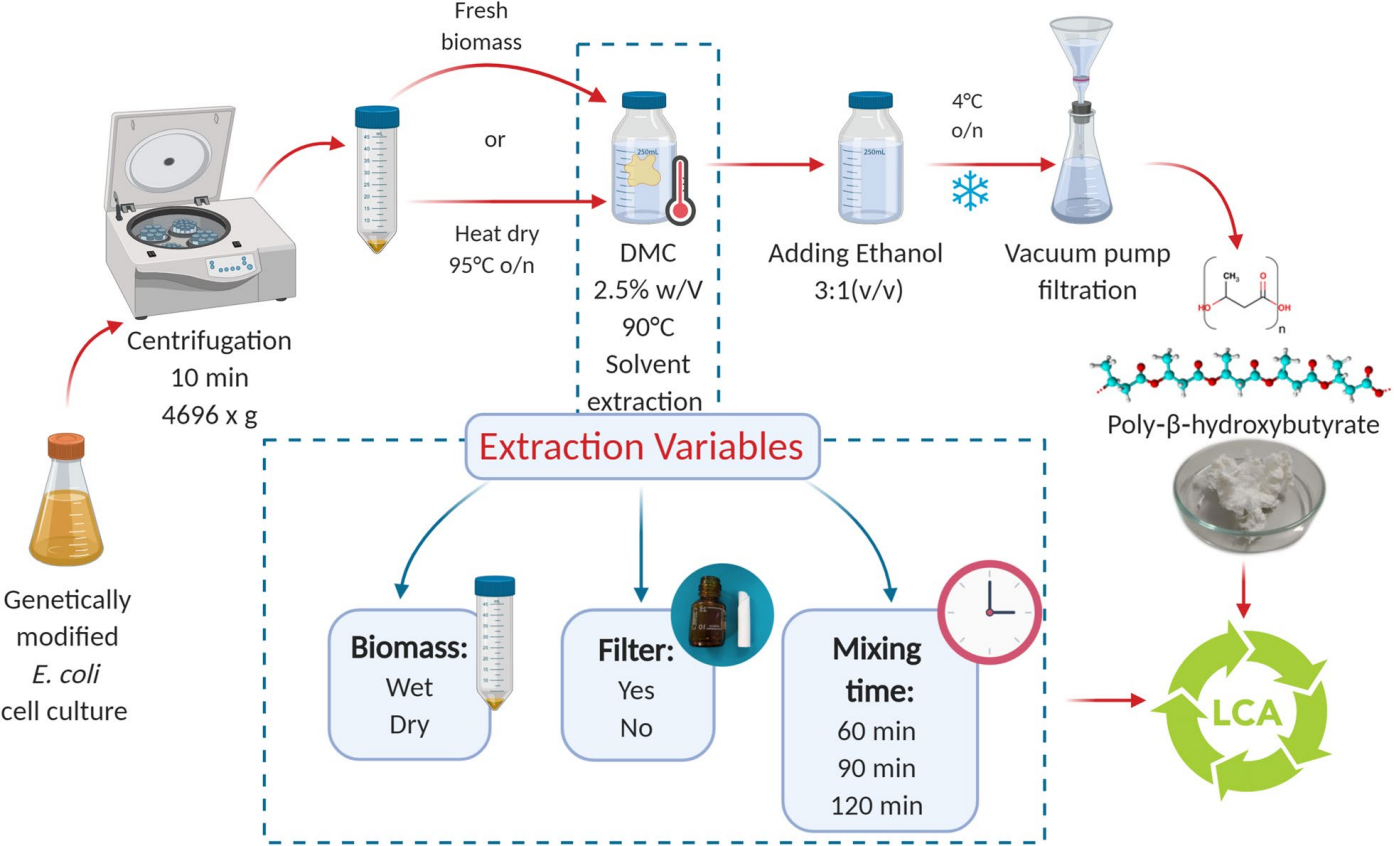
Schematic representation of experimental set-up of the present study. *E. coli* biomass is treated with DMC to extract the PHB: the extraction protocol varies on the base of the selected experimental variables (biomass, filter, mixing time). A life cycle assessment of the extraction process based on DMC/ethanol is performed and compared to that of chloroform/hexane extraction. Created with http://www.Biorender.com

The present study investigates the application of DMC on PHB-rich biomass by varying the extraction procedure on the base of three factors: biomass, filter and mixing time (Fig. 1). In this study we evaluated the extraction efficiency of DMC on both pre-treated (i.e. dry) and untreated biomass (i.e. wet). Biomass pre-treatments, such as heating, freeze-drying, sonication or chemical oxidation reduce the integrity of microbial cell membrane, in favour of an incremented PHB extraction yield [13, 21]. However, pre-treating the biomass can affect the physical properties of the polymer as for chemical oxidation, which is well known to decrease the molecular weight of the polymer [22] as well as for lyophilization and heating which cause polymer chains rearrangement with a consequent variation of its crystallinity [23]. Furthermore, the application of those pre-treatments also requires an investment in additional material, operational energy, and costs. By the introduction of a paper filter, we attempted to: (1) improve the purity of the PHB without affecting its properties, (2) improve the manageability of the extraction procedure in a lab-scale context. Indeed, the filter was initially conceived as a physical barrier between biomass and solvent phase to enable a tidy separation of the two in a small volume of reaction and achieve highly pure PHB [15]. However, the evidence of our experiments did not support the filter application, but rather highlighted the efficacy of using wet biomass instead of the dry one. The mixing time, defined as the time of contact between biomass and solvent, is the third variable we decided to investigate. The selection of the optimal mixing time is a critical point for solvent-based extraction of PHB as showed by Fiorese et al. [24]. Here the importance of mixing time was also analysed in relation to the biomass and the filter with regard to PHB extraction yield, purity, and molecular properties.

In order to address all the purposes of the present study, a genetically modified *Escherichia coli* strain hosting PHB biosynthetic pathway was selected. The application of an engineered *E. coli* rather than a natural PHB-producing bacterium is reliant on the fact that *E. coli* is a well-known microorganism whose metabolism and cultivation are characterized in detail. *E. coli* is easy to handle, it can ensure fast growth rates despite the application scale, it guarantees high yield of biomass and PHB accumulation [25–28]. Moreover, *E. coli* is a Gram-negative like most of the natural PHB producers (e.g. *Cupriavidus necator or Azotobacter vinelandii*) with which it shares the same cellular membrane structure. Finally, it is worthwhile noting that, unlike natural PHB-producing strains, *E. coli* does not own depolymerase activity, which makes it a valid candidate for being a model microorganism for PHA recovery study.

In our research, the extraction protocol was adapted to the laboratory scale and conditions, which are quite different from the industrial approach. Many studies reported the extraction procedure in a lab-scale frame, but only few investigated the life cycle of the DMC [29]. Recently, the chemical industry has shown an increased interest in Life Cycle Assessment (LCA). However, much uncertainty still exists about the environmental impacts of solvents, especially in lab-scale setups [30]. For this reason, each experiment conducted in this study was accompanied by an LCA analysis. A complete scheme of the design at the base of our study is available as Fig. 1. If on the one hand, the present work addresses the optimization of DMC-based extraction to maximize the PHB recovery and purity, on the other hand, it aims at assessing the impact at many levels of DMC applied as green solvent a priori. In conclusion, this study traces the guidelines to select the optimal conditions for a more sustainable PHB extraction.

## Results and discussion

### PHB extraction yield and purity

**Fig. 2:**
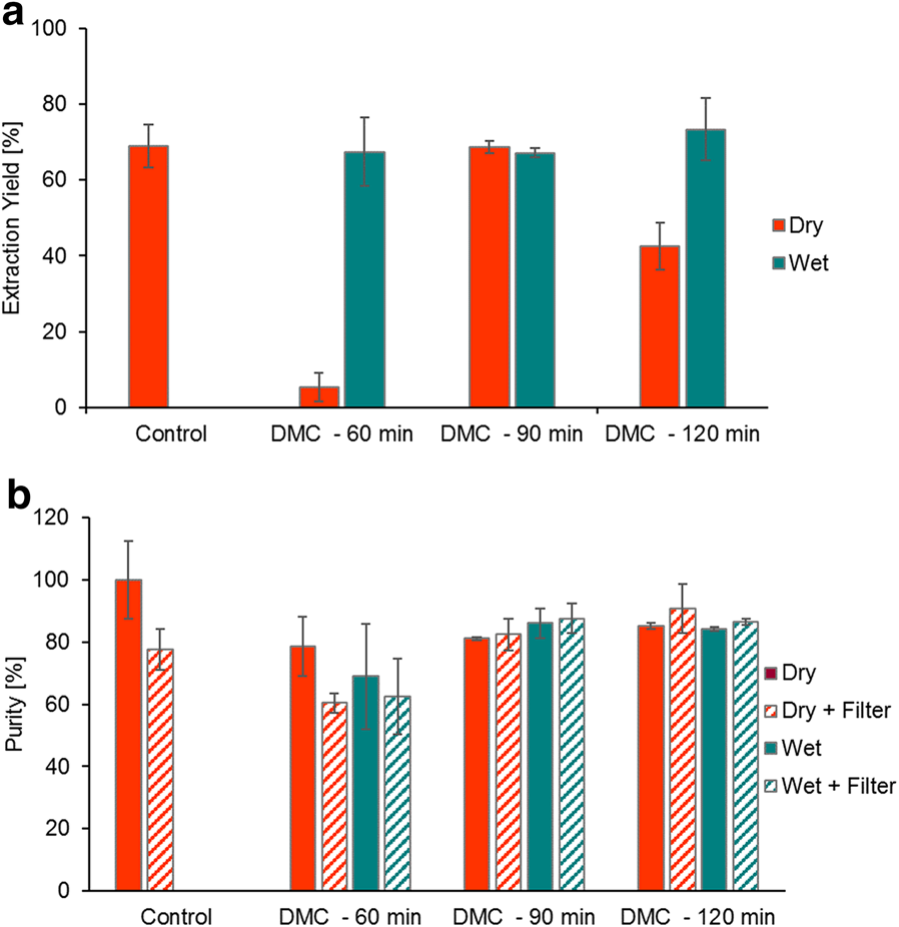
PHB extraction yield (**a**) and purity (**b**) of samples extracted by chloroform as the standard method (control) and by DMC at different mixing times. Dry and wet biomass are in red and green, respectively; the presence of the filter is indicated by the dashed bars

Results show that DMC-based extraction can compete with the standard method based on chloroform (Fig. 2).

In terms of extraction yield, the DMC-based extraction is comparable to that based on chloroform when the mixing time is equal to 90 min both on wet and dry biomass (Fig. 2a). When processing wet biomass, the extraction yields assume always values above 67%, independently on the mixing time. Mixing time seems to acquire specific importance when the DMC extraction occurs on dry biomass. Moreover, a contact time of 90 min is optimal, resulting in a rise of the extraction yield (68.67 ± 1.66%). Table 1 compares the extraction yields of this work and other publicly available studies.

**Table 1:** PHB extraction yield depending on the extraction process

Biomass	Extraction process	Extraction yield (%)	References
Dry	Chloroform	39 ± 2	[11]
Dry^a^	Dimethyl formamide, solid–liquid extraction	68 ± 4	[11]
Lyophilized	1,2-Propylencarbonate, 120 °C for 15 min	75	[31]
Wet	Butyl acetate, 103 °C, for 30 min	96 ± 1	[14]
Lyophilized	Dimethyl carbonate, 90 °C for 60 min	20 ± 1	[32]
Dry	Dimethyl carbonate, 90 °C for 90 min	69 ± 2	This study
Wet	Dimethyl carbonate, 90 °C for 120 min	73 ± 8	This study
Dry	Chloroform, 60 °C for 120 min	69 ± 6	This study

^a^Pre-treated with ethanol

Aramvash et al. [14] recorded an extraction yield of 96% using butyl acetate on wet biomass for a contact time of 30 min (Table 1). Similar values were also found in McChalicher et al. [31] and Manangan and Shawaphun [11], using propylene carbonates and dimethyl formamide as solvents, respectively (Table 1), but with a very short mixing time concerning the former. Our results indicate that a contact time of 60 min is not enough for extracting the PHB from dry biomass, with a final yield of 5.3 ± 3.7%. The same extraction protocol, except for the lyophilized biomass and the application of a mixed culture, is applied in Samorì et al. [32] who reported a PHB extraction yield of 20 ± 1% (Table 1). Such difference in results could depend not only on the mixing time, but also on the initial biomass state (lyophilized or dried), and the utilization of different PHB-producing strains. On the other hand, a mixing time of 120 min on dry samples, results to be excessive since the extraction yield drops to 42.5 ± 6.2%. Although not showed here, results of the extraction yield in presence of the filter are available at Additional file 1: Table S1 and are discussed in the next paragraph. Concerning the PHB purity (Fig. 2b), chloroform extraction gave a value of 100 ± 12.5%, which drop to 88.3 ± 6.7% when adding the filter. The PHB purity values are nearly similar in DMC-based extraction at 90 and 120 min, regardless of the biomass and filter (Additional file 1: Table S1). Even though the use of a paper filter should simplify the separation between polymer and biomass and favour PHB collection [15], the results collected in this study (Fig. 2 and Additional file 1: Table S1) do not support the use of the filter as real improvement in the purification process in the specific frame of our work. A possible explanation could be found in the different application scale of Fei et al. [15] and the present study, large and small scale, respectively. Another point might be the leading forces involved in PHB separation, i.e. pressure and gravity in Fei et al. [15] with respect to the sole diffusion through the filter pores toward the boiling solvent. As a matter of time, the purity decreases when biomass and solvent are mixed for 60 min, which may indicate that a shorter mixing time is less incisive on impurities breakup than longer contact time [24]. However, considering the high standard deviation, this variation is not statistically significant, and it cannot be associated with the wet state of the biomass with certainty.

### Combined effect of experimental factors

The individual and combined effect of the investigated experimental variables—paper filter, pellet, and mixing time—on the extraction yield was evaluated through regression analysis.

**Fig. 3:**
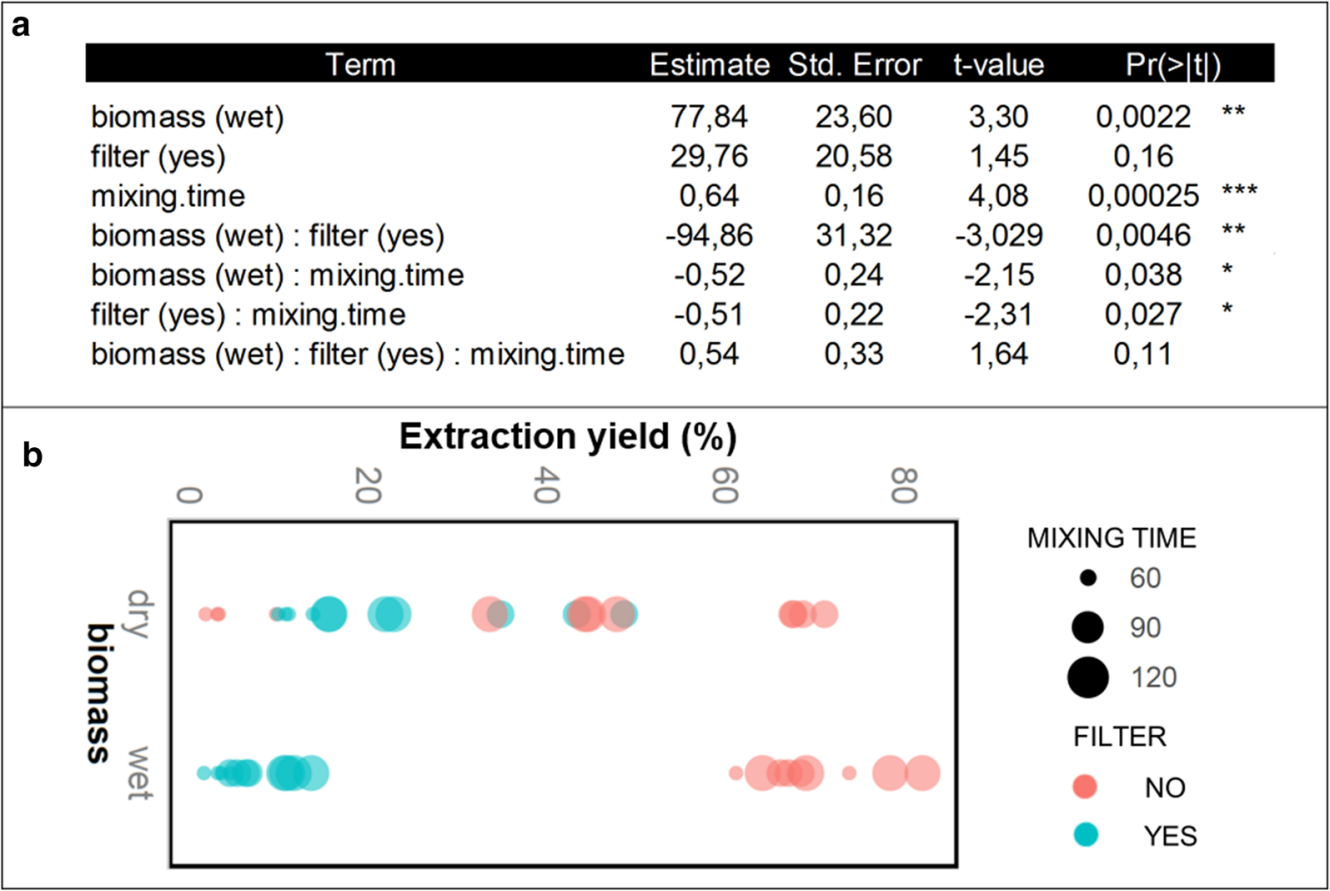
Combined effect of experimental factors. **a** Regression analysis for modelling the relationships between the response variable (extraction yield) and the explanatory variables (paper filter, mixing time and pellet) was conducted by fitting a regression model through the Generalized Additive Models for Location, Scale and Shape (GAMLSS) package in the R statistical computing environment. The distribution for the response variable in the GAMLSS was selected from the normal family of distributions. The table shows the partial regression coefficients and standard errors of a regression model inclusive of interaction terms, the associated *t*-statistics and *P*-values. **b** Shown is the decreased extraction yield associated with the usage of wet pellet and the presence of the paper filter by mixing time

Wet pellet and increased mixing time show a statistically significant positive influence on extraction yield, whereas no statistically significant dependence of PHB extraction yield on the filter was detected (*α* = 1%), as shown in Fig. 3a. The impact of experimental factor combinations involving the filter did not turn out to be statistically significant except for the interaction of the filter with pellet, which is highlighted in the data clustering displayed in Fig. 3b. Indeed, combining filter with wet pellet corresponds to the cluster associated with the lowest yield, whereas the usage of wet pellet and the removal of the paper filter correspond to the cluster associated with the highest extraction yield. This observation can reflect the fact that the barrier action exerted by the filter is augmented when the biomass is wet, likely owing to the hydrophilic properties of the filter, which becomes a sponge in contact with water. When the biomass is dry, the filter pores may be more available to the solvent transit. Moreover, in the presence of filter, stirring was not possible, so that the only energy allowing the interaction between solvent and biomass was generated by keeping the temperature close to the solvent boiling point.

### PHB characterization

#### Differential scanning calorimetry (DSC) and thermal gravimetric analysis (TGA)

The thermal properties of the purified PHB are reported in Table 2 and illustrated in Additional file 2: Figure S1. The degradation temperature (*T*_d_), which was found to be around 300 °C, is thus comparable between samples extracted by chloroform and by DMC. In contrast, the glass transition temperature (*T*_g_) tends to increase, in particular as a consequence of using wet biomass. The melting temperature (*T*_m_) of the control sample is about 4 and 6 °C below that of the samples obtained by DMC-based extractions. A good agreement with values reported in the literature is found [14, 15, 24]. Pronounced differences were found in the melting enthalpy (Δ*H*_m_) and crystallinity (degree of crystallinity) when comparing the solvents used. Interestingly, the differences were found to depend also on the biomass state. It is known that the physical properties of a biopolymer rely on the polymer molecular weight, which in turn depends on the applied extraction procedures [14, 24, 33]. More precisely, in vivo PHB chains are stabilized by a significant concentration of water content, around 10–15%, which forms hydrogen bonds with the carbonyl groups of the biopolymer, forming a pseudo-crosslink [23, 34]. However, pre-treatment steps such as lyophilization or air-drying, expose the polymer to excessive energy, causing a decrease of water content. The consequences are the rearrangement of PHB chains and a change in polymer crystallinity [23]. Therefore, the enthalpy of crystallization (Δ*H*_c_), which reflects the energy applied per gramme of produced crystal, varies in the same way. As a result, the extraction efficiency is affected too [31]. In conclusion, the higher extraction efficiency observed in wet biomass could depend on a better interaction between the solvent and the PHB branches.

**Table 2:** DSC thermal properties of PHB extracted with chloroform (control) and DMC for a mixing time of 90 min, from dry and wet biomass

Solvent	Mixing time (min)	Biomass State	*T*_d_^a^ (°C)	*T*_g_^b^ (°C)	*T*_c_^c^ (°C)	Δ*H*_c_^d^ (J/g)	Degree of crystallinity^e^ (%)	*T*_m_^f^ (°C)	Δ*H*_m_^g^ (J/g)	References
Chloroform at 60 °C	120	Dry	289	2.2	68.2	60.4	41	164	104	This work
DMC at 90 °C	90	Dry	287	3.5	64.1	76.7	53	168	141	This work
DMC at 90 °C	90	Wet	287	4.5	63.2	43.8	30	170	83.2	This work
Acetone/ethanol/propylene carbonate at 110 °C		Wet						175	88.3	[15]
Butyl acetate				4.8	41			176		[14]
1,2-Propylene carbonate at 100 °C	45	Wet		4.9			60	176	84.5	[24]
1,2-Propylene carbonate at 100 °C	15	Wet		4.8			57	172	80.5	[24]

The filter was present in all cases

*T*_g_, glass transition temperature; *T*_c_, crystallization temperature; Δ*H*_m_, melting enthalpy; Δ*H*_c_, crystallization enthalpy; *C*_r_, the degree of crystallization; *T*_m_, melting temperature

#### Attenuated total reflectance infrared (ATR)

**Fig. 4:**
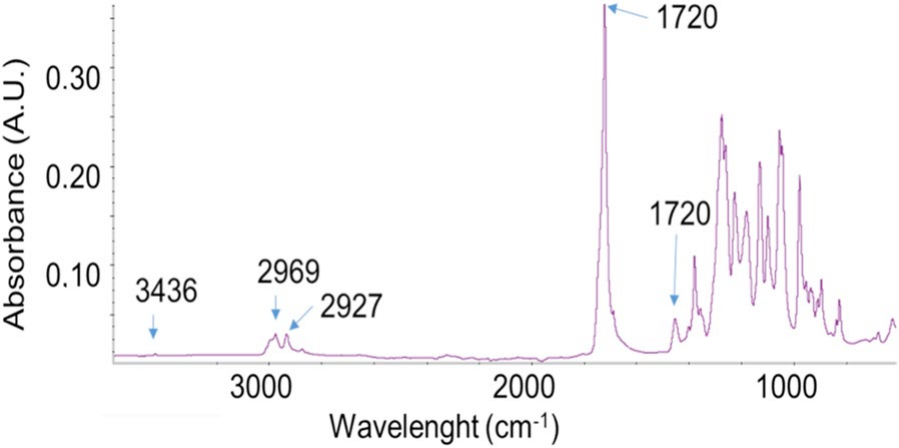
ATR infrared analysis of PHB sample extracted by dry biomass with DMC/ethanol for 120 min. 1720 cm^−1^ ν_CO_; 3436 cm^−1^ ν_CO_ overtone; 1452 cm^−1^ *δ*a_CH2_; *δ*a_CH3_ 2969 and 2927 cm^−1^ ν_CH3_

The IR spectrum of a sample represents its total chemical composition where every chemical compound in the sample makes its own distinct contribution to the absorbance spectrum. The total spectrum is determined by the chemical structure of each component and the degree to which each component contributes, directly related to the concentrations of the components of the sample. Thus, it is possible to have an idea of impurities present in the samples. The main impurities for PHB are cellular proteins, lipids, fatty acids in the ester form and phosphodiester from backbone of nucleic acids (DNA and RNA) or phosphorylated proteins/polyphosphate storage products [35, 36]. A second type of impurities is determined by the chemical compounds used for the purification themselves. The main adsorption bands of PHB are presented in Fig. 4.

The band at 1,720 cm^−1^ corresponds to the ester carbonyl group stretching (ν_CO_) typical of PHB, lipids and fatty acids while the overtone at 3436 cm^−1^ it is characteristic of PHB and other PHA [37, 38]. The band at 1452 cm^−1^ is related to the asymmetric bending of –CH_2_ or –CH_3_ (δ_CH2_; δ_CH3_) present in PHB and proteins [39]. The bends at 2969 and 2927 cm^−1^ indicate the presence of an alkyl –CH_3_ group.

It is possible to evidence the main impurities from their characteristic bands, e.g. the presence of proteins can be evidenced by the Amide I and Amide II bands at, respectively, about 1650 cm^−1^ and 1540 cm^−1^. DNA, RNA and another polyphosphate can be evidenced from the symmetric stretching P=O (ν_PO_) at 1080 cm^−1^ [40]. Spectra of the other representative samples are reported in the Additional file 2: Figure S2.

#### Molecular weight

The molecular weights (MW) of PHB extracted by using DMC and chloroform are reported in Table 3 and compared on the basis of equal mixing time.

**Table 3:** Average values of the MW of the extracted PHB at the different experimental conditions

Extraction	Mixing time (min)	Biomass	PHB MW (MDa)
Chloroform/hexane	120	Dry	0.35
DMC/ethanol	120	Dry	0.23
DMC/ethanol	120	Wet	1.1

The outcome of viscosimeter analysis strictly depends on the constant value used in the Mark–Houwink equation (Eq. 3), which has been selected in line with the experimental conditions applied in this study. The molecular weights of PHB presented herein, are consistent with literature data: MW of PHB ranges between 0.5 and 20 × 10^6^ Da when feeding recombinant *E. coli* with glucose [41–43]. Besides that, PHB extracted from wet biomass using DMC is about 1 MDa, while lower values, i.e. 0.23 and 0.35 MDa, are recorded for dry biomass extracted with DMC and chloroform, respectively. It may appear that the absence of water favours a molecular degradation of the polymer chains, which could consequently affect the extraction yield. Although a clear explanation to this regard is not yet demonstrated, similar data were described for lyophilized cells of *Cupriavidus necator* exposed to 100° and 120 °C in cyclohexanone [44].

### LCA analysis

Prior studies have noted the importance of the LCA application in biopolymers production [45, 46]. Therefore, this study assessed the potential lifecycle environmental impacts of PHB extraction based on DMC compared with chloroform. As it has been explained in the LCI section, two PHB production scenarios were assessed: one based on chloroform/hexane and the other on DMC/ethanol. In line with Righi et al. [47], our analysis revealed dry pellet without the application of filter to be the best solutions in terms of LCA and for this reason the relative LCA results listed for each ILCD impact categories are shown in Table 4.

**Table 4:** Comparative characterization LCA results per 1 g of PHB produced from DMC/ethanol (dry biomass) or from chloroform/hexane

Impact category	Abbreviation	Unit	PHB from DMC/ethanol	PHB from chloroform/hexane
Climate change	CC	kg CO_2 eq_	5.34·10^2^	2.09·10^2^
Ozone depletion	OD	kg CFC-11 eq	1.37·10^–5^	3.65·10^–2^
Human toxicity, non-cancer effects	HTNC	CTUh	1.84·10^–5^	2.49·10^–5^
Human toxicity, cancer effects	HTC	CTUh	2.45·10^–6^	3.47·10^–6^
Particulate matter	PM	kg PM_2.5 eq_	1.81·10^–1^	9.07·10^–2^
Ionizing radiation in human health	IRHH	kBq U^235^_eq_	8.29·10^0^	1.09·10^1^
Ionizing radiation in ecosystems	IRE	CTUe	6.40·10^–5^	7.90·10^–5^
Photochemical ozone formation	POF	kg NMVOC _eq_	1.66·10^0^	5.14·10^–1^
Acidification	AC	molc H^+^_eq_	2.47·10^0^	8.87·10^–1^
Terrestrial eutrophication	TE	molc N _eq_	4.23·10^0^	1.26·10^0^
Freshwater eutrophication	FWE	kg P _eq_	8.80·10^–2^	8.72·10^–3^
Marine eutrophication	ME	kg N _eq_	2.67·10^–1^	1.15·10^–1^
Freshwater ecotoxicity	WT	CTUe	2.13·10^2^	5.63·10^1^
Land use	LU	kg C _deficit_	1.36·10^2^	1.40·10^2^
Water resource depletion	WRD	m^3^ H_2_O _eq_	1.49·10^2^	1.85·10^2^
Mineral, fossil and renewable resource depletion	FFD	kg Sb _eq_	1.11·10^–2^	7.73·10^–3^

**Fig. 5:**
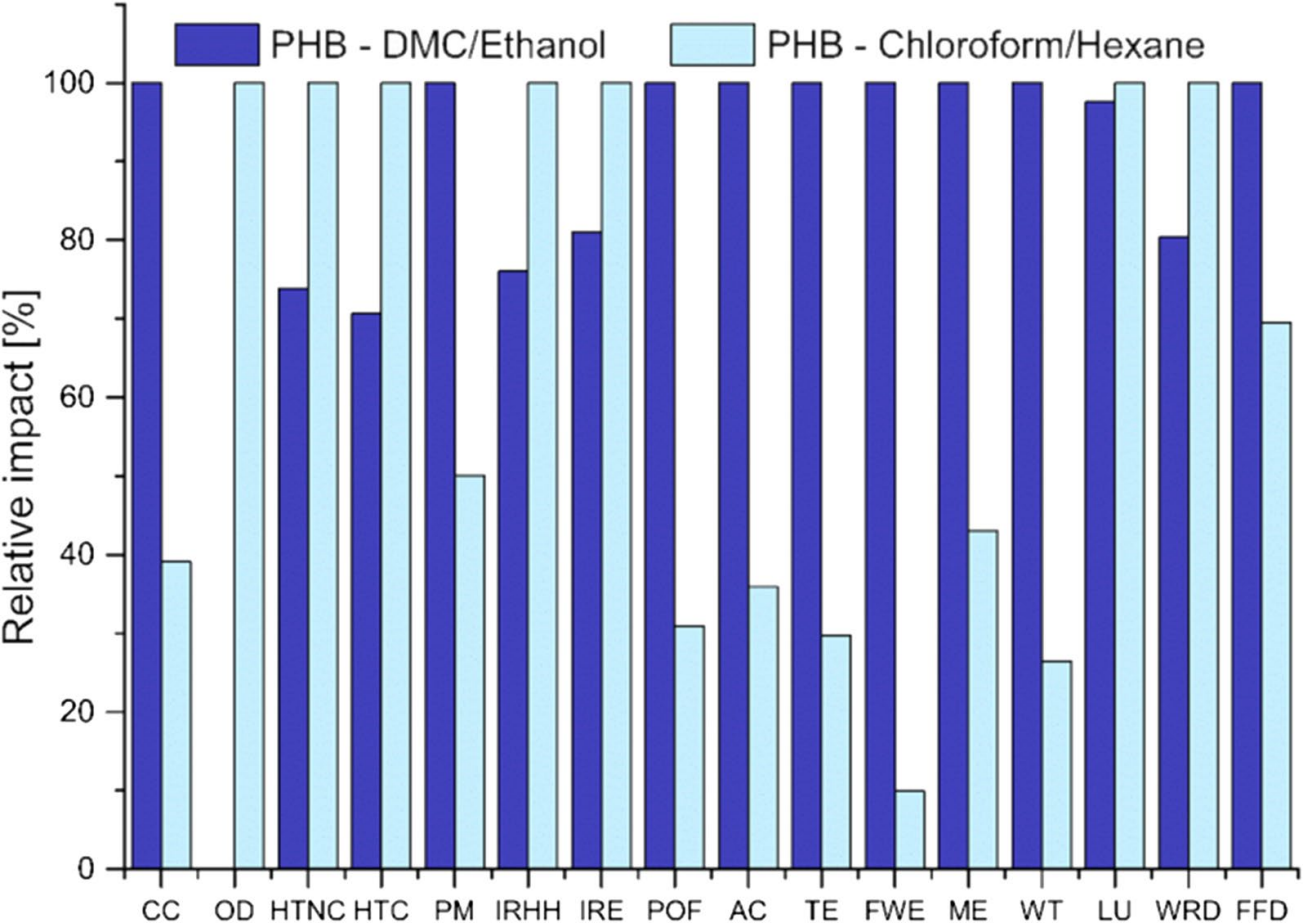
Comparative LCA results of PHB production for the two assessed routes. DMC/ethanol or chloroform/hexane as solvent/antisolvent

To accurately represent impacts with different units in the same graphic, Fig. 5 provides the environmental impacts in relative percentage.

**Fig. 6:**
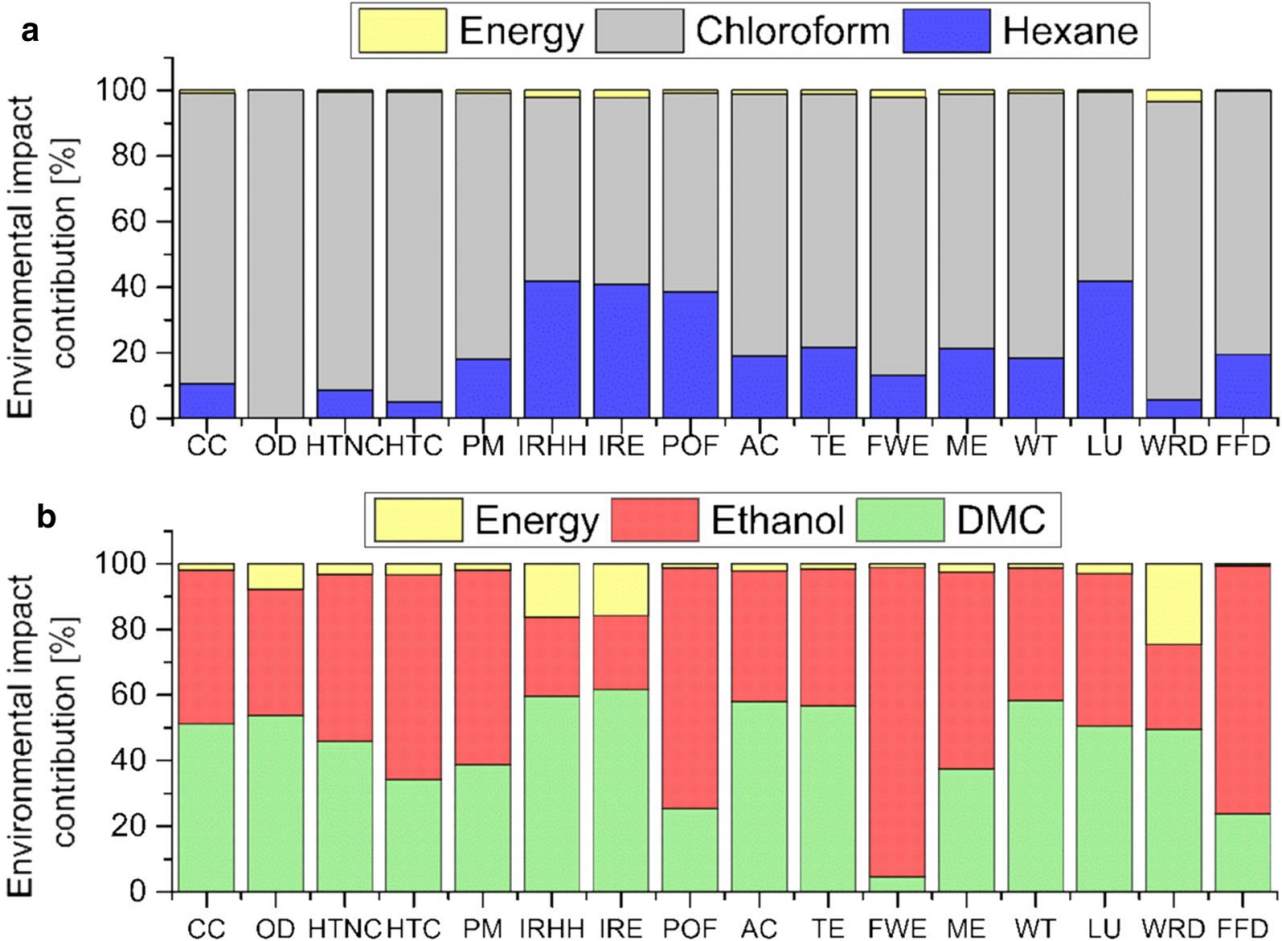
Environmental contributions (%) of 1 g of PHB. **a** PHB produced with chloroform and hexane. **b** PHB produced with DMC and ethanol as solvent and antisolvent

The comparative results for 1 g of PHB produced through two different techniques showed that PHB extraction from DMC/ethanol got better environmental results in OD, HTNC, HTC, IRHH, IR, LU and WRD. The remaining impact categories assessed revealed better environmental behaviour for the PHB extraction based on chloroform/hexane. In order to determine the main contributing phases for each impact category, a contribution analysis was included. Figure 6 shows the environmental contributions of the items in the PHB extraction process in both assessed systems.

The lowest influence of energy is represented by the chloroform/hexane case study (Fig. 6) since a minor amount of solvent is used to get a similar amount of PHB and since the enthalpy of chloroform vaporization is lower than that of DMC. Globally, the chloroform in the chloroform/hexane extraction route has higher influence on the environmental profile than DMC. In fact, the case of DMC/ethanol extraction the environmental contribution is almost equally distributed between the two chemicals. This fact means that acting on chloroform will lower the environmental impact of the system easily. However, in order to reduce the environmental impacts generated by the DMC/ethanol extraction, both compounds have to be adjusted. Ozone depletion (OD) represents the destructive effects on the stratospheric ozone layer over a time horizon of 100 years. The contribution of DMC/ethanol case study to this category is halved compared to that of chloroform/hexane extraction route. Comparative toxic unit for humans (CTUh) is an indicator which estimates the increase in morbidity in the total human population per unit of mass of emitted chemical [48]. Human toxicity, cancer and non-cancer effects, ionizing radiation on human health (IRHH) and ecosystems (IRE) got better results in DMC/ethanol case study compared to the chloroform/hexane one. As shown in Fig. 6a, this is because of the significant contribution of chloroform to this category. The same tendency was observed for land use (LU) and water resource depletion (WRD) indicators. One of the most known environmental categories is climate change (CC) which represents global warming potential in kg of CO_2_-equivalents. This indicator favoured the chloroform/hexane extraction rather than the DMC/ethanol application. It is a general opinion that DMC represents a promising substitute to conventional chlorinated solvents because of their lower human toxicity. However, a deeper analysis highlights the drawbacks of using DMC in other environmental categories, such as fossil fuel depletion (FFD), water toxicity eutrophication (WTE), acidification (AC) (10 of 16 environmental categories). Notable is also the level of affection associated to the use of ethanol on other relevant categories (FEW, POF and FFD). Hence, replacing ethanol with other less impacting compounds would change the impact on these or more categories in support of the DMC/ethanol extraction route (Figs. 5 and 6b). LCA analysis related to biopolymers shows a large variation among environmental results. The fossil polymer with the lowest overall life cycle impacts is polypropylene. Published works agree that PHAs, including PHB performed better in CC and FFD categories compared with conventional fossil-based polymers [46]. This is because the studies that reported lower CC impact values accounted for carbon uptake during biomass growth or using waste stream [49]. Nevertheless, to our knowledge, there are no studies in literature that consider the same boundary conditions. Hence, it is difficult to give balanced reading of our results. In summary, his examination gave rise to divergent issues which should be considered for further process optimization and unbiased analysis of the results.

## Conclusions

The multifaceted exploration of DMC-based PHB extraction herein reported extends the knowledge of the variables affecting PHB recovery. Among these variables, mixing time significantly influenced the extraction yield of PHB. Physical and molecular properties changed depending on the biomass state and solvent/antisolvent choice. Wet biomass generates PHB with low crystallinity grade and high molecular weight. Despite the well-known potential of chloroform/hexane-based extraction, only slight differences in PHB extraction yields were observed when compared to DMC/ethanol application. According to our LCA analysis, DMC could be a promising substitute to chloroform. Indicators like human toxicity and ionizing radiation, ozone depletion and water resources depletion show optimal values when DMC/ethanol is applied. Nevertheless, other relevant environmental categories have a minor impact when using chloroform/hexane extraction compared to DMC/ethanol one. In disagreement with similar studies, the findings of the present work do not completely support the application of DMC as a *green solvent.* However, the choice of the antisolvent can modify the level of impact on the examined categories. Despite the relatively limited lab-scale investigation, this work offers novel and valuable insights into PHB extraction process, including environmental aspects not discussed so far.

## Methods

### Biomass cultivation

The bacterial strain used in this study was kindly supplied by the group of Dr. Auxiliadora M. Prieto (CSIC, Spain). It is a genetically modified *E. coli* BL21 (DE3), hosting a plasmid with the three genes devoted to the PHB biosynthetic (*phb ABC*) coming from *Ralstonia eutropha* H16 and a chloramphenicol resistance, which are activated on glucose [50]. Prior to begin the experimental campaign, the biomass production by the engineered *E. coli* was evaluated. For each flask filled with 250 mL of working volume and supplemented with 10 gL^−1^ of glucose, about 2.3 g of biomass containing 54% of PHB were ensured after 12 h of growth approximately. Hence, the described cultivation technique was applied to the whole experimental trial. The strain, stocked at − 20 °C in 15% v/v glycerol, was cultured in Luria–Bertani (LB) medium supplemented with chloramphenicol (20 µg/mL) and glucose (10% v/v). Each culture was prepared in Erlenmeyer flasks filled with around 40% of culture broth. To maximize the biomass growth, pre-cultures of 70 mL and culture of 250 mL flask were prepared, respectively. A volume of 1.5 mL of *E. coli* glycerol stock was transferred to the preculture flask and incubated for 12 h, at 37 °C and 200 rpm (ES-20/60, Orbital Shaker-Incubator, Biosan) [51]. After it, a pre-culture volume was transferred to fresh LB medium to have an initial optical density at 600 nm (OD_600_) of 0.3, in a culture volume of 250 mL. A volume of 250 mL containing biomass were distributed in 50 mL falcon tubes and centrifuged two times at 4696×*g* for 15 min, to collect the cell pellet (SL 16R Centrifuge, Thermo Fischer Scientific, Germany). The obtained pellet was washed with deionized H_2_O and centrifuged at 4696×*g* for 15 min. Depending on the selected extraction process, the wet cell pellet was directly treated or dried in the oven at 105 °C for 12 h and ground by a ceramic mortar and pestle.

### Chloroform/hexane-based extraction

As the reference method [15], the PHB was extracted by treating dry biomass with 99% chloroform/ 95% hexane, as a solvent/antisolvent couple, respectively. The solvent was added to the dry pellet in a volumetric ratio of 15:1, stirred at 60 °C for 120 min. In the case of filter-mediated extraction, wet or dry biomass was previously wrapped in a paper filter with a pore size of 11 μm, arranged as a tea-bag container (Whatman grade 1). At the end of the reaction the entire solution was centrifuged at 4696×*g* for 5 to 10 min to remove the pellet. When the filter was present, the centrifugation step was not necessary. PHB precipitation was obtained by the addition of hexane, used as antisolvent because of its miscibility with the solvent. The combination of the two causes a change of solvent mixture properties which reduces the bio-polymer solubility and generates supersaturation followed by a rapid crystallization [15, 52, 53].

95% hexane was added to chloroform in a volumetric ratio of 1:3. Hence, the solvent:antisolvent mixture was left at 4 °C for 24 h, after which the precipitated PHB was separated from hexane with vacuum filtration and finally air-dried.

### Dimethyl carbonate/ethanol-based extraction

**Fig. 7:**
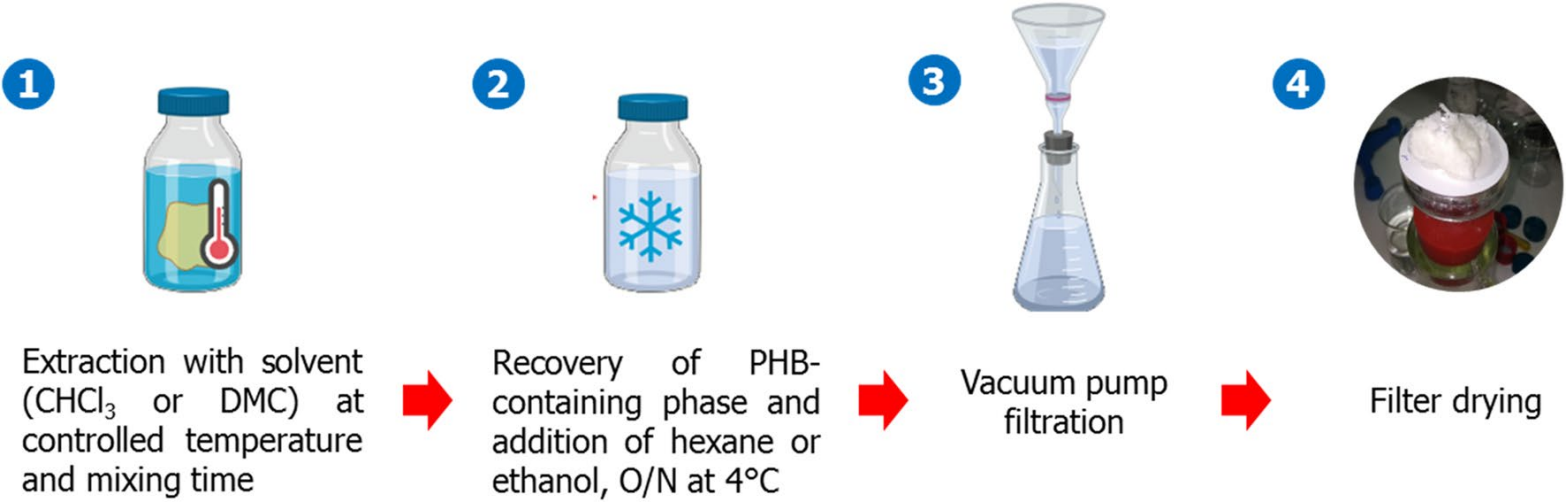
Extraction phases: (1) the solvent reacts for a certain time with the biomass which is in the form of a pellet, (2) an antisolvent is added to precipitate the PHB and left overnight at 4 °C, (3) the solution is filtered via vacuum, (4) the PHB is dried on paper filter. Created with http://www.Biorender.com

The PHB extraction with 99% DMC and ethanol is based on a modified protocol by Samorì et al., [32] consisting of different reaction mixing time, pellet texture and filter use (Fig. 7).

In case of direct solvent-biomass extraction, the wet or dry pellet was put directly in 20-mL glass bottles, where DMC was added to the collected biomass respecting the ratio 2.5% (w/v). The reaction was set under continuous stirring at 90 °C, for to 60, 90, 120 min depending on the experimental run. At the end of each extraction, the solution was transferred into 50-mL falcon and centrifuged at 4696×*g* for 10 min, to separate the organic phase hosting the PHB from the biomass. Therefore, the organic phase was recovered, and pure ethanol was added at a DMC:ethanol volumetric ratio of 1:3 to enable PHB precipitation. The mixture was stored for 12 h at 4 °C. The PHB was then filtered via vacuum pump and air-dried. In the case of filter-mediated extraction, wet or dry biomass was in a paper filter arranged as a tea-bag container (Whatman grade 1). Hence, there was no need to centrifuge the samples to separate the PHB-rich organic phase from the exhaust biomass, but just PHB precipitation was required via ethanol supplement.

### PHB quantification

To verify the specific amount of PHB produced by genetically modified *E. coli* cells during each test, every time a new culture was set up a sample corresponding to 5 mL of final culture broth was kept and stored at -20 °C for successive analysis of the cell dry weight and of the correspondent PHB amount. Cell dry weight was obtained gravimetrically. The sample collected was centrifuged at 18,816×*g* for 5 min, washed two times and dried at 80 °C for 12 h in an oven. Once estimated the correspondent biomass concentration (g L^−1^), 5 mg of biomass were hydrolysed by 1 mL of 96% H_2_SO_4_ under stirring of 800 rpm, into an agitated oil bath at 90 °C for one hour. At the end of the reaction, a dilution of 1:1000 was done, and the samples were analysed by HPLC equipped with a column ROA-organic acid H+ (8%) (Phenomenex) and a photodiode array detectors (PDA) under an isocratic flux of 0.7 mL min^−1^ of 5 mM of H_2_SO_4_ and a temperature of 50 °C. The PHB-related peak was revealed at 210 nm with an elution time of 23.09 min, which corresponds to the crotonic acid (the monomer coming from the PHB hydrolysation) used as a reference standard [54]. The PHB yield was estimated on the base of Eq. 1:

1 $${\text{PHB extraction yield}}\left( \% \right) = \frac{{{\text{purified PHB}}\left( {\text{g}} \right)}}{{{\text{PHB present in the treated biomass}}\left( {\text{g}} \right)}}.$$

### PHB purity estimation

The purity of the recovered PHB was investigated by the HPLC method above described. Representative samples of the extracted PHB were ground into a ceramic mortar, hydrolysed by H_2_SO_4_ (ground and hydrolysed biomass) and quantified as follows (Eq. 2):

2 $${\text{PHB purity}}\left( \% \right) = \frac{{{\text{HPLC quantified of extracted PHB}} \left( {\text{g}} \right) }}{{{\text{ground and hydrolysed PHB}} \left( {\text{g}} \right)}}.$$

### Experimental design of the extraction process

To explore the potential of using DMC-based extraction, rather than chloroform, the following design of experiments (DoE) was conceived. Three experimental variables, namely *mixing time*, *pellet* and the presence of *filter* were accounted for (Table 5). The *mixing time* experimental variable could assume three discrete levels (60, 90, 120 min), while the categorical *pellet* and *filter* data types assumed binary values, dry/wet and yes/no, respectively.

**Table 5:** Text matrix for the DoE

Run	Mixing time (min)	Pellet	Filter
1	90	Dry	No
2	120	Dry	No
3	60	Dry	Yes
4	60	Dry	No
5	90	Dry	Yes
6	120	Dry	Yes
7	90	Wet	Yes
8	60	Wet	Yes
9	120	Wet	Yes
10	90	Wet	No
11	120	Wet	No
12	60	Wet	No

### Data analysis

Regression analysis for modelling the relationships between the response variable (extraction yield) and the explanatory variables (mixing time, pellet and filter) was conducted by fitting a regression model inclusive of the interaction terms through the Generalized Additive Models for Location, Scale and Shape (GAMLSS) package in the R statistical computing environment [55, 56]. The distribution for the response variable in the GAMLSS was selected from the normal family of distributions. Significance of the regression coefficients resulting from model fitting was evaluated by comparing the ratio of coefficients estimates and their standard errors with a t distribution (*α* = 0.01).

### Differential scanning calorimetry (DSC) and thermogravimetric analysis (TGA)

DSC was applied to study the thermal transition properties of the PHB extracted by the standard chlorinated solvent compared to the DMC-based extraction (DSC 204 F1 Phoenix® Netzsch). The analysis was led on both wet and dry biomass extracted for 90 min which is the mixing time reporting the highest PHB yield. The decomposition temperatures of the extracted polymer were determined by TGA, at 10 °C/min (Mettler Toledo TGA/SDTA 851e system). The studies were performed under an Argon flux of 50 mL min^−1^, scanning a temperature range going from 25 to 500 °C.

### Attenuated total reflection (ATR)

Fourier transform infrared (FTIR) spectroscopy (Bruker Tensor II) was performed on the materials in attenuated total reflection (ATR) configuration. The spectra were acquired accumulating 32 scans in 4000–600 cm^−1^ range with resolution of 2 cm^−1^.

### Determination of PHB molecular weight

The average molecular weight determined through a viscosimeter analysis [11, 57] made by a Cannon-Fenske tube (100 mL size) at a temperature of 29.3 °C (FungiLab). Before performing the quantification, purified polymers were dissolved in pure chloroform at 50 °C under gentle stirring in a closed vial, for being filtered through a qualitative filter paper for technical use (Whatman grade 1575) and cast on a 50 mL Becker. Hence, the obtained PHB dry films were dissolved in chloroform at 30 °C in a closed vial at a concentration of 0.5% w/w and poured in the viscometer, which was located in a thermostat and incubated 10 min before to perform the analysis. The PHB solution flow time was measured, and successive measurements were made on PHB serial dilutions. The molecular weight of standard PHB (Goodfellow) used as reference was 5.7E + 05 g mol^−1^. Intrinsic viscosity (*η*) and relative final molecular weights were extrapolated by Mark–Houwink relationship (Eq. 3), using the following experimental formula reported [57]:

3 $$\eta = 1.18 \times 10^{ - 4} M_{{\text{w}}}^{0.78} .$$

From each measurement the relative (*η*_r_), specific (*η*_sp_) and reduced viscosity (*η*_red_) were calculated, following the reported equations, where the concentration (*C*) and *η*_red_ are expressed in g mL^−1^ and mL g^−1^_,_ respectively:

4 $$\eta_{{\text{r}}} = \frac{{\eta_{{{\text{sol}}}} }}{{\eta_{{{\text{solv}}}} }},$$

5 $$\eta_{{{\text{sp}}}} = \frac{{\eta_{{\text{sol }}} - \eta_{{{\text{solv}}}} }}{{\eta_{{{\text{solv}}}} }},$$

6 $$\eta_{{{\text{red}}}} = \frac{{\eta_{{\text{sp }}} }}{C}.$$

### Life cycle assessment method

According to ISO 14040-44 [58, 59] an LCA includes four stages: (i) goal and scope definition, (ii) life cycle inventory, (iii) life cycle impact assessments and (iv) interpretation of the results.

(i) Goal and scope stage consists of the identification of the LCA purpose, defining the functional unit, the sources of data, the boundaries of the analysis and the additional tools used in the LCA. A cradle-to-gate approach has been chosen for the LCA study. The goal was to analyse the influence of the solvent/antisolvent used in PHB extraction. Initial biomass amount, solvent, antisolvent and the enthalpy of vaporization were the items included in the assessment of PHB production. Therefore, the environmental results will be got per gramme of PHB production.
(ii) Life cycle inventory (LCI) consists of the quantification of the inputs and outputs associated with the functional unit. Typically, inventory data include raw materials and energy consumption, and the emission of solid, liquid, and gaseous wastes.
  To quantify the environmental impacts of the extraction process, it was selected the standard extraction with chloroform and hexane and the PHB production with DMC and ethanol.
  In this research work, the LCI gathers solvent, antisolvent, energy to evaporate the solvent (enthalpy) and PHB. DMC enthalpy (0.194  kWh/kg) was reported by Breil et al. [60] and chloroform enthalpy (0.069  kWh/kg) by Holbrook [61]. This energy to vaporize the solvent was supposed to be part of the system as a low-voltage power source. The energy needed to vaporize chloroform is lower than DMC. Regarding the database, Ecoinvent 3.4 was implemented in the internationally acknowledged SimaPro 8 software to build the LCA. It is not always possible to find every single chemical compound in the common LCA databases. Hence, missing items are often modelled according to stoichiometry or information reported in the literature. In the present work, DMC was modelled according to Righi et al. [62]. To be as replicable and transparent as possible, the dataset has been declared. Dataset names from Ecoinvent database used to conduct the comparative LCA are shown in Table 6.
(iii) Life cycle impact assessment (LCIA) consists in the quantification of the effects of the phases defined in the LCI. It foresees the selection of impact categories, firstly, and classification, secondly. It means that the elementary flows from the life cycle inventory were assigned to impact categories according to the ability of substances to contribute to different environmental problems. Subsequently, in the characterization stage, the impact of each emission or resource consumption is modelled quantitatively, according to the environmental assessment approach. The result is an impact score in a common unit to all contributions within the impact category by applying the so-called characterization factors (e.g. kg CO_2_-equivalents for greenhouse gases contributing to the impact category *Climate Change*). For this purpose, there are several methods. The selection of impact categories is performed in line with the goal and scope definition of the LCA assessment [63]. The present study follows the International Reference Life Cycle Data System (ILCD) handbook methodology, developed by the European Joint Research Centre (JRC) [64]. This implementation corresponds to the ILCD version 1.0.9 of May 2016 [65]. This LCIA method includes 16 midpoint impact categories: Climate Change (CC), Ozone Depletion (OD), Human Toxicity, Non-Cancer effects (HTNC), Human Toxicity, Cancer effects (HTC), Particulate Matter (PM), Ionizing Radiation HH (human health) (IRHH), Ionizing Radiation E (ecosystems) (IRE), Photochemical Ozone Formation: (POF), Acidification (AC), Terrestrial Eutrophication (TE), Freshwater Eutrophication (FEW), Marine Eutrophication (ME), Freshwater Ecotoxicity (WT), Land Use (LU), Water Resource Depletion (WRD), Mineral, fossil and renewable resource depletion (FFD)
(iv) The interpretation phase of an LCA is the last step in every LCA study. It consists in the interpretation of LCI and LCIA results with the purpose of extracting relevant observations for further developments of a single process or significant conclusions in the case different processes have been compared.

**Table 6:** Life cycle inventory of PHB extraction process based on experimental results

	PHB from DMC/ethanol (run 11)			PHB from chloroform/hexane (standard extraction)		
	Dataset	Amount	Unit	Dataset	Amount	Unit
Output	PHB	0.235	g	PHB	0.239	g
Inputs	Biomass	0.546	g	Biomass	0.55	g
	DMC (modelled)	23.01	g	Hexane {GLO}| market for | Alloc Def, U	15.84	g
	Ethanol, without water, in 99.7% solution state, from ethylene {RER}| ethylene hydration | Alloc Def, U	50.89	g	Trichloromethane {GLO}| market for | Alloc Def, U	11.92	g
	Electricity, low voltage {Europe without Switzerland}| market group for | Alloc Def, U	4.463·10^–03^	kWh	Electricity, low voltage {Europe without Switzerland}| market group for | Alloc Def, U	8.177·10^–04^	kWh

Dataset from Ecoinvent database are reported in the table

## Supplementary Information

**Additional file 1: Table S1.** Raw values corresponding to each experimental run (total 12).

**Additional file 2: Figure S1.** DSC curves of PHB samples. **A)** Chloroform/Hexane (dry biomass); **B)** DMC/Ethanol for 90 min (dry biomass); **C)** DMC/ Ethanol for 90 min (wet biomass). The filter was applied to all three extractions. **Figure S2.** ATR spectra of PHB samples (1–6). Sample 1: PHB extracted from dry biomass with Chloroform/Hexane for 120 min. Sample 2 and 3: PHB extracted with DMC/Ethanol for 90 min from dry and wet biomass, respectively. No filter was applied in sample 1–3. Sample 4: PHB extracted from dry biomass with Chloroform/Hexane for 120 min. Sample 5 and 6: PHB extracted with DMC/Ethanol for 90 min from dry and wet biomass, respectively. Filter was used in sample 4, 5, 7. The presence of amide I and amide II bands in sample 1 and 2 evidence the presence of proteins. There is no evidence of impurities from ATR analyses in sample 3 and 4. A band at about 798 cm^−1^ may indicate the presence of some DMC residual in sample 5 and 6.

## Data Availability

All data generated or analysed during this study are included in this published article and its additional material files.

## Abbreviations

AC: Acidification
ATR: Attenuated total reflectance infrared
CC: Climate change
CTUh: Comparative toxic unit for humans
DMC: Dimethyl carbonate
DSC: Differential scanning calorimetry
FEW: Freshwater eutrophication
FFD: Mineral, fossil and renewable resource depletion
HTC: Human toxicity cancer effects
HTNC: Human toxicity non-cancer effects
ILCD: International Reference Life Cycle Data System
IRE: Ionization radiation ecosystems
IRHH: Ionizing radiation on human health
JRC: European Joint Research Centre
LCA: Life Cycle Assessment
LCI: Life Cycle Inventory
LCIA: Life Cycle Impact Assessment
LU: Land use
ME: Marine eutrophication
MW: Molecular weight
OD: Ozone depletion
PHAs: Polyhydroxyalkanoates
PHB: Poly-3-hydroxybutyrate
PM: Particulate matter
POF: Photochemical ozone formation
*T*_c_: Crystallization temperature
TE: Terrestrial eutrophication
*T*_g_: Glass transition temperature
TGA: Thermogravimetric analysis
*T*_m_: Melting temperature
WRD: Water resource depletion
WT: Freshwater toxicity
Δ*H*_c_: Crystallization enthalpy
Δ*H*_m_: Melting enthalpy
