# Supplementary material for: Novel insights in dimethyl carbonate-based extraction of polyhydroxybutyrate (PHB)
Source: Biotechnol Biofuels. 2021 Jan 7;14:13. doi: 10.1186/s13068-020-01849-y (PMC7792028; doi:10.1186/s13068-020-01849-y)

Additional file 1

**Novel insights in dimethyl carbonate-based extraction of polyhydroxybutyrate (PHB)**

**Table S1.** Raw values corresponding to each experimental run (total 12).


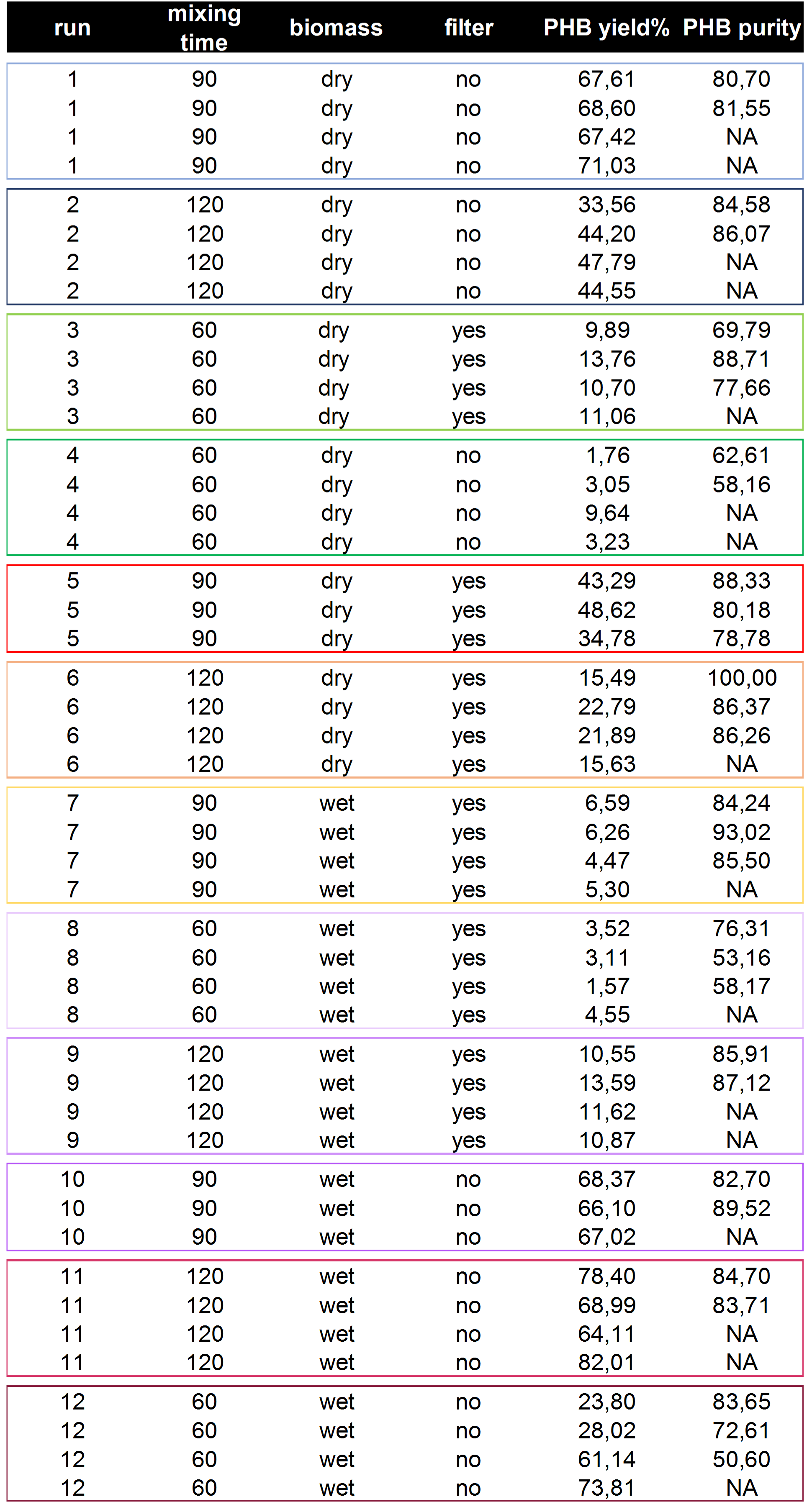

Supplement: Supplementary file 1 — Additional file 1: Table S1. Raw values corresponding to each experimental run (total 12). [file 13068_2020_1849_MOESM1_ESM.docx]
