# Supplementary material for: Novel insights in dimethyl carbonate-based extraction of polyhydroxybutyrate (PHB)
Source: Biotechnol Biofuels. 2021 Jan 7;14:13. doi: 10.1186/s13068-020-01849-y (PMC7792028; doi:10.1186/s13068-020-01849-y)

Additional file 2

**Novel insights in dimethyl carbonate-based extraction of polyhydroxybutyrate (PHB)**

**Figure S1.** DSC curves of PHB samples. **A)** Chloroform/Hexane (dry biomass) ; **B)** DMC/Ethanol for 90 minutes (dry biomass); **C)** DMC/ Ethanol for 90 minutes (wet biomass). The filter was applied to all three extractions.


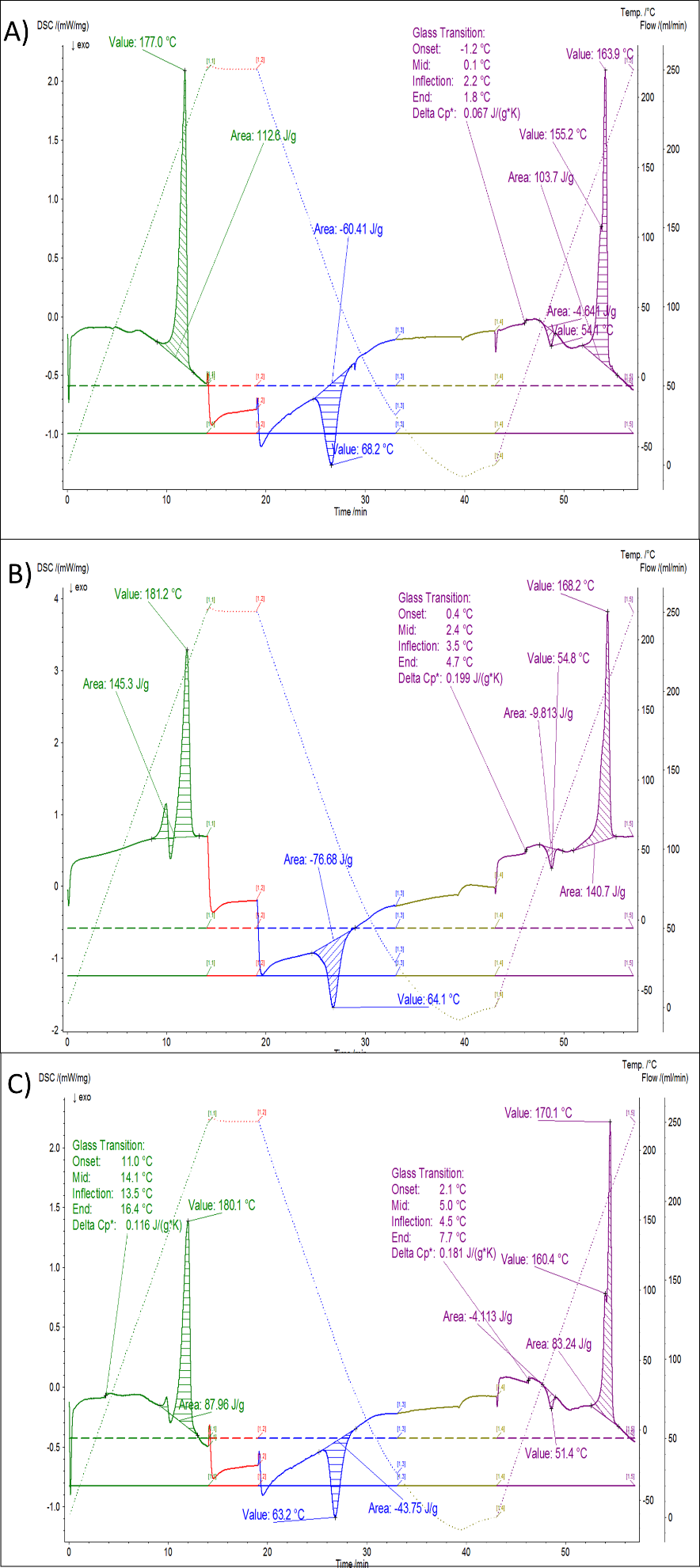


**Figure S2.** ATR spectra of PHB samples (1-6). Sample 1: PHB extracted from dry biomass with Chloroform/Hexane for 120 minutes. Sample 2 and 3: PHB extracted with DMC/Ethanol for 90 minutes from dry and wet biomass respectively. No filter was applied in sample 1-3. Sample 4: PHB extracted from dry biomass with Chloroform/Hexane for 120 minutes. Sample 5 and 6: PHB extracted with DMC/Ethanol for 90 minutes from dry and wet biomass respectively. Filter was used in sample 4, 5, 7. The presence of amide I and amide II bands in sample 1 and 2 evidence the presence of proteins. There is no evidence of impurities from ATR analyses in sample 3 and 4. A band at about 798 cm^-1^ may indicate the presence of some DMC residual in sample 5 and 6.


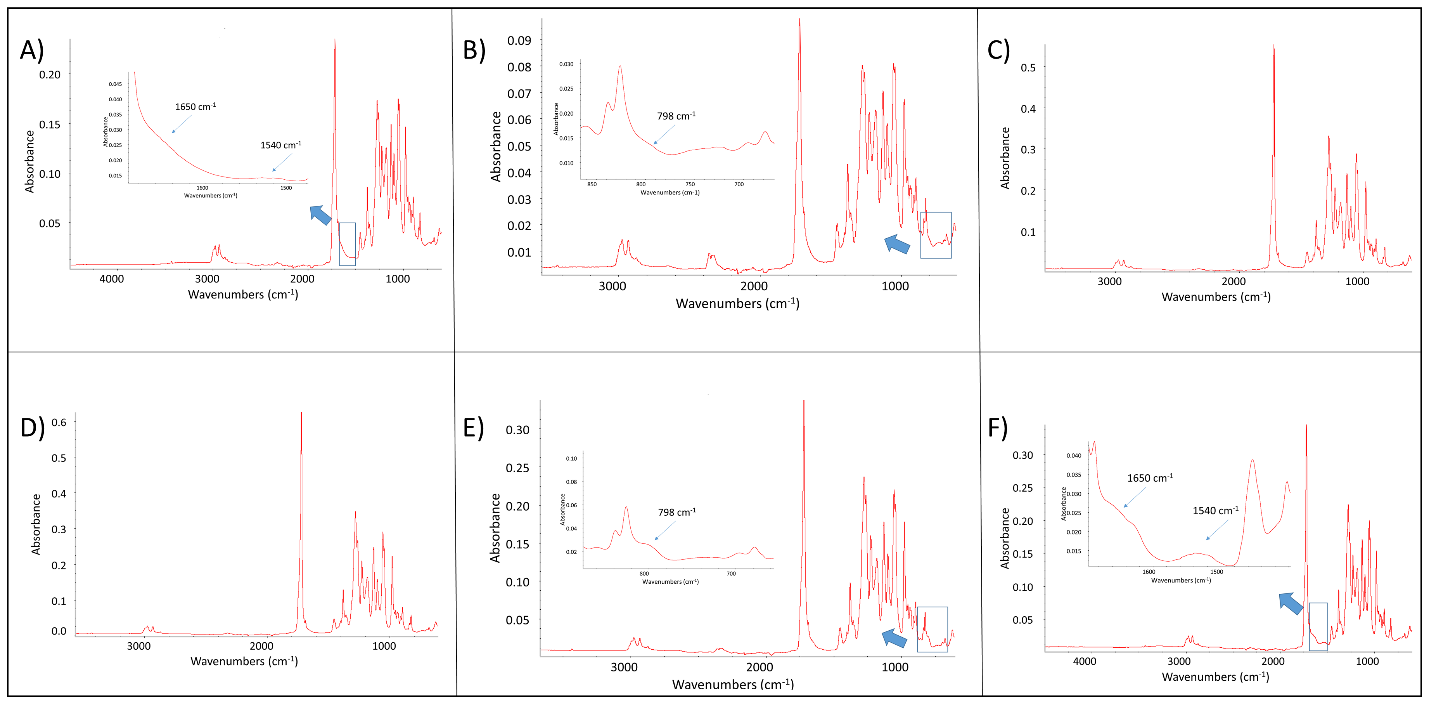

Supplement: Supplementary file 2 — Additional file 2: Figure S1. DSC curves of PHB samples. A) Chloroform/Hexane (dry biomass); B) DMC/Ethanol for 90 min (dry biomass); C) DMC/ Ethanol for 90 min (wet biomass). The filter was applied to all three extractions. Figure S2. ATR spectra of PHB samples (1–6). Sample 1: PHB extracted from dry biomass with Chloroform/Hexane for 120 min. Sample 2 and 3: PHB extracted with DMC/Ethanol for 90 min from dry and wet biomass, respectively. No filter was applied in sample 1–3. Sample 4: PHB extracted from dry biomass with Chloroform/Hexane for 120 min. Sample 5 and 6: PHB extracted with DMC/Ethanol for 90 min from dry and wet biomass, respectively. Filter was used in sample 4, 5, 7. The presence of amide I and amide II bands in sample 1 and 2 evidence the presence of proteins. There is no evidence of impurities from ATR analyses in sample 3 and 4. A band at about 798 cm−1 may indicate the presence of some DMC residual in sample 5 and 6. [file 13068_2020_1849_MOESM2_ESM.docx]
